# Increased CD103^−^CD8^+^ TILs with TPEX phenotype replenish anti-tumor T cell pool in mismatch repair-proficient CRC

**DOI:** 10.1007/s00262-025-04214-w

**Published:** 2025-11-03

**Authors:** Jinsheng Liu, Jintao Zeng, Ting Zhou, Mi Wu, Xiufang Weng

**Affiliations:** 1 Department of Gastrointestinal Surgery, Shengli Clinical Medical College of Fujian Medical University, Fujian Provincial Hospital, Fuzhou University Affiliated Provincial Hospital, Fuzhou, 350001 Fujian China; 2 Department of Colorectal Surgery, the First Affiliated Hospital of Fujian Medical University, Fuzhou, 350005 Fujian China; 3 Department of Immunology, School of Basic Medicine, Tongji Medical College, Huazhong University of Science and Technology, Wuhan, 430030 China; 4 Department of Rheumatology and Clinical Immunology, Jiangxi Provincial People’s Hospital, The First Affiliated Hospital of Nanchang Medical College, Nanchang, 330006 China

**Keywords:** Colorectal cancer, CD103^−^CD8^+^ T cells, T_PEX_ phenotype, TCR clonotypes, Anti-PD-1treatment

## Abstract

While mismatch repair-deficient (dMMR) colorectal cancers (CRCs) exhibit strong immunogenicity and better response to immune checkpoint inhibitors, the more prevalent mismatch repair-proficient (pMMR) CRCs typically show poor T- cell infiltration and inferior outcomes. Profiling the limited tumor-infiltrating CD8^+^ T cells helps identify responders and guides new strategies to enhance their infiltration and function in pMMR CRC tumors. Our study reveals that the proportion and number of CD103^−^CD8^+^ T (CD103N) cells are significantly increased in pMMR CRC tissue compared to adjacent non-tumor tissue. Distinguish from CD103^+^CD8^+^ T (CD103P) cells with elevated T_EX_ markers, these CD103N cells display a precursor exhausted T cells (T_PEX_) phenotype with elevated stemness properties, reduced exhaustion markers, and retained functional capacity to secrete anti-tumor mediators. Moreover, CD103N cells in pMMR CRC shared a substantial number of identical TCR clonotypes with both CD103P cells in tumor and CD103N cell in peripheral blood. In dMMR CRC patients, enrichment of T_PEX_-like CD103N cells is associated with a favorable prognosis following anti-PD-1 therapy, suggesting their association with clinical outcome. Our findings identify an expanded population of CD103N cells exhibiting a T_PEX_ phenotype with anti-tumor potential in pMMR CRC, highlighting their promise as therapeutic targets for recruitment into the tumor microenvironment to enhance the efficacy of immunotherapy.

**Supplementary Information:**

The online version contains supplementary material available at 10.1007/s00262-025-04214-w.

## Introduction

Colorectal cancer (CRC) ranks third in global incidence and second in mortality. In China, the incidence of CRC has been increasing annually due to changes in lifestyle and dietary habits [1, 2]. While surgery remains a potentially curative option for early-stage CRC, radiotherapy, chemotherapy, and targeted therapies generally fall short of achieving a cure [3]. The emergence and advancement of immunotherapy have brought new hope for treating non-resectable CRC, making curative outcomes increasingly attainable [4]. However, the heterogeneity and complexity of the CRC tumor microenvironment (TME) pose significant challenges to the advancement of immunotherapies.

CRCs can be categorized into two distinct groups based on their mismatch repair (MMR) status: those that are proficient MMR (pMMR), which may exhibit either microsatellite stability (MSS) or low-level microsatellite instability (MSI-L), and those with deficient MMR (dMMR) that display high-level microsatellite instability (MSI-H) [5, 6]. dMMR CRCs tend to elicit a stronger immune response due to their increased antigenicity and higher mutation burden, which leads to the production of more neoantigens. In contrast, pMMR tumors have fewer neoantigens and are therefore less immunogenic. These tumors typically have a -T- cell-inflamed microenvironment, making them susceptible to PD-1/PD-L1 blockade. However, pMMR CRCs are more prevalent, accounting for approximately 85% of all CRC cases, and are generally associated with a poorer prognosis compared to their dMMR counterparts [7]. Although pMMR CRC is recognized as a “cold tumor”, - tumor-infiltrating lymphocytes (TILs), particularly CD8^+^ cytotoxic T cells, are also observed and associated with better prognosis and response to immune checkpoint inhibition therapy [8, 9]. Profiling these T-cells not only stratifies potential responders but also drives the development of novel combinatorial approaches to boost T cell infiltration and function in pMMR tumors.

Previously studies have shown that the intensity of tumor-infiltrating T cells within tumor tissues is closely associated with tumor progression and favorable patient outcomes following treatment [10, 11]. Among these TILs, CD8^+^ T cells, also known as cytotoxic T cells, are the most powerful anti-tumor effectors within the tumor immune microenvironment. CD8^+^ T cells recognize antigens presented by antigen-presenting cells or tumor cells via MHC-I molecules through their *αβ* T-cell receptor (TCR), thereby initiating activation and clonal expansion. Activated CD8^+^ T cells exert their cytotoxic effects through multiple mechanisms, including the secretion of anti-tumor cytokines such as IFN-*γ* and TNF-*α*, the release of cytotoxic mediators such as perforins and granzymes, or the direct killing of tumor cells via the FASL-FAS signaling pathway [12]. CD8^+^ T cells in the intestinal epithelium and lamina propria have been shown to exhibit a tissue-resident memory (T_RM_) phenotype, characterized by the markers CD103 (*ITGAE*) and CD69 [13]. Notably, CD8^+^ T_RM_ cells represent a substantial proportion of tumor-reactive T cells in various solid tumors, including non-small cell lung cancer [14], head and neck squamous cell carcinoma [15], melanoma [16], and CRC [17]. Elegant mouse models have also demonstrated that CD103^+^ T_RM_ cells can differentiate from CD103^−^CD8^+^ T cells [18]. However, the distribution, phenotype, and functional differences between these two populations in CRC, as well as their potential for interconversion and their impact on CRC prognosis, remain poorly understood.

In a harsh immunosuppressive microenvironment, characterized by dysfunctional immune cells and abnormal metabolites, CD8^+^ T cells gradually evolve into terminally exhausted T cells (T_EX_) under chronic and persistent stimulation [19, 20]. This results a loss of their capacity to maintain effective anti-tumor immunity. Studies have shown that tumor-infiltrating CD8⁺ T cells undergo distinct stages of exhaustion, which can be broadly categorized into two major paths [21]. One subset differentiates into precursor exhausted T cells (T_PEX_), which retain self-renewal capacity and sustain the exhausted T cell pool. The other subset acquires cytolytic effector functions as effector T cells, expressing markers such as granzyme B and PD-1, and subsequently progresses toward a terminally exhausted state (T_EX_), characterized by highly exhaustion level and limited proliferative potential [22]. T_PEX_ cells exhibit classical memory cell characteristics, such as pro-longed, self-renewal capacity, and developmental plasticity [23], which are characterized by high expression of TCF1, SELL, and SLAMF6, while expressing low levels of exhaustion markers like TIM3, CD39, and CD101 [24–26]. Under TGF-*β* and IL-2 signaling, the transcription factor BCL6 antagonizes PRDM1, inhibiting the transition of T_PEX_ cells to a T_EX_ phenotype and enhancing CD8^+^ T cell-mediated anti-tumor immunity during anti-PD-1 therapy [27].

In the current study, we found that CD103^−^CD8^+^ T (CD103N) cells were significantly increased in pMMR CRC tissues, which exhibited predominant Tpex phenotype and retained anti-tumor capacity. This population provides potential target for improving cell infiltration and enhancing immunotherapy efficacy.

## Materials and methods

### Patients and samples

This study utilized samples obtained from 38 proficient mismatch repair (pMMR) colorectal cancer (CRC) patients and 37 age-matched healthy donors. From CRC patients, we collected peripheral blood, paired non-tumor adjacent tissue (sampled ≥ 2 cm from the tumor edge), and tumor tissue. Patient characteristics are detailed in Supplementary Table 1. Peripheral blood samples from the healthy donors served as controls. All participants were recruited from Tongji Hospital (Wuhan, China) and Fujian Provincial Hospital (Fuzhou, China). Patients were enrolled based on the following criteria: (i) histopathologically confirmed local colorectal adenocarcinoma with pMMR status; (ii) absence of recent systemic infection or history of immune system diseases. Written informed consent was obtained from all participants prior to enrollment.

### Study approval

The ethical review board of Tongji Medical College, Huazhong University of Science and Technology (Hubei, China) approved the protocols for this research, ensuring adherence to established ethical standards. This study was conducted with an Ethics ID of 2023S063.

### Preparation of single-cell suspension

Peripheral blood samples were obtained using heparin sodium collection tubes. Peripheral blood mononuclear cells (PBMCs) were separated through gradient centrifugation with a 1.077 g/ml Ficoll solution (TBDscience, Cat: LTS1077, China) at room temperature (25 °C) and a speed of 2000 rpm for 20 min.

Freshly harvested colorectal tissues were promptly dissociated, including the following steps for the isolation of mononuclear cells (MNCs). The muscular layer of the colorectal tissue was carefully excised with tissue scissors and the remaining tissue was then cut into small fragments. These fragments were washed with phosphate-buffered saline (PBS) containing 200 units ml^−1^ of penicillin–streptomycin (New Cell & Molecular Biotech Co.,Ltd, Cat: C100C5, China). The washed tissue pieces were digested in 1 mM Dithiothreitol (Beyotime, Cat: ST043-5 g, China) (DTT)/PBS for 15 min at 37 °C to remove the mucus layer, then rinsed twice with cold PBS. The tissue was processed by two steps as following: 1) digesting in a 1 mM ethylenediaminetetraacetic acid (EDTA)/PBS solution at 37 °C for 30 min on a shaker set to 250 rpm. The digested mixture was then passed through a 70 µm pore size cell strainer (Biosharp, Cat: BS-70-XBS, China), and the supernatant was centrifuged at 400 g for 10 min to get intraepithelial cells. 2) The remaining tissue pieces were further digested with a solution containing 0.1 mg/mL Liberase (Sigma-Aldrich, Cat: 05401020001, St Louis, MO, USA) and 0.2 mg/mL DNase I (Sigma-Aldrich, Cat: 10104159001) at 37 °C for an additional 30 min for extraction of lamina propria cells. Tumor - infiltrating cells were isolated by merging cells obtained from above two steps. Finally, intraepithelial lymphocytes (IELs) from intraepithelial cells, lamina propria lymphocytes (LPLs) from and tumor-infiltrating lymphocytes (TILs) were separated by gradient centrifugation using a 1.077 g/mL Ficoll solution (TBDscience, Cat: LTS1077) at 800 g for 20 min at room temperature.

### Surface staining and flow cytometry detection

To assess the proportion and phenotype of CD8⁺ T cells, mononuclear cells (MNCs) isolated from peripheral blood or colorectal tissue were subjected to surface staining and analyzed by flow cytometry. A total of 1 × 10^6^ cells were incubated with Human TruStain FcX™ (Fc receptor blocker; BioLegend, Cat: 422302, San Diego, CA, USA) at 25 °C for 15 min to minimize non-specific binding. Cells were then stained with the indicated fluorochrome-conjugated antibodies, diluted in PBS containing 1% fetal bovine serum (FBS), and incubated at 25 °C for 30 min. After washing, stained cells were analyzed using a FACSVerse (BD Biosciences) or Cytek™ Aurora (Cytek). The antibodies used for surface staining included: anti-CD3-APC-Cy7 (UCHT1, BioLegend, Cat: 300426), anti-CD8A-BV510 (SK1, BioLegend, Cat: 344732), anti-CD69-PE (FN50, BioLegend, Cat: 310906), anti-CD69-FITC (FN50, BioLegend, Cat: 310904), anti-CD45-AF700 (HI30, BD, Cat: 560566), anti-CD4-PE (RPA-T4, BD, Cat: 562658), anti-CD103-BB515 (K041E5, BD, Cat: Ber-ACT8), anti-CD39-PerCP-eFluor 710 (eBioA1, eBioscience, Cat: 46–0399-42), anti-CD57-BV421 (NK-1, BD, Cat: 568894), anti-PD-1-BV421 (29F.1A12, BioLegend, Cat: 135218), anti-PD-1-PE (A17188B, BioLegend, Cat: 621608) and anti-TIM3-AF647 (7D3, BD, Cat: 565558).

### Intracellular cytokine staining analysis

To evaluate the cytokine secretion capacity of CD8⁺ T cells, intracellular staining for IFN-*γ*, TNF-*α*, and IL-2 was performed. Cells (1 × 10^6^) were cultured in 96-well V-bottom plates and stimulated with 25 ng/mL phorbol 12-myristate 13-acetate (PMA, Cat: 16,561–29-8) and 500 ng/mL ionomycin (ION, Cat: 56,092–81-0) (both from Sigma-Aldrich) for 30 min. Following stimulation, 3 μg/mL Brefeldin A (Invitrogen, Cat: 00–4506-51, Carlsbad, CA, USA) was added, and the cells were incubated for an additional 3.5 h at 37 °C in a 5% CO_2_ incubator to block cytokine secretion. After stimulation, cells were collected and initially stained with surface markers, including anti-CD3-APC-Cy7 (UCHT1, BioLegend, Cat: 300426) and anti-CD8A-BV510 (SK1, BioLegend, Cat: 344732). Subsequently, the cells were fixed and permeabilized using Fixation/Perm diluent (Invitrogen), followed by intracellular staining. Antibodies used for cytokine detection included: anti-IFN-*γ*-FITC (4S.B3, BioLegend, Cat: 502506), anti-TNF-*α*-BV650 (MAb11, BD, Cat: 563418), anti-IL-2-PerCP-eFluor 710 (MQ1-17H12, eBioscience, Cat: 46–7029-42), anti-granzyme A (CB9, BioLegend, Cat: 507220) and anti-perforin (dG9, BioLegend, Cat: 308106).

### Co-culture of HCT116 cells with PBMCs

HCT116 cells were treated with 10 μg/mL mitomycin C (MedChemExpress, Cat: HY-13316, China) for 3 h, followed by co-culture with CellTrace Violet (CTV, Biolegend, Cat: 425101)-labeled PBMCs from healthy donors in 24-well plates. Each well was seeded with 3 × 10^4^ HCT116 cells and 6 × 10^5^ PBMCs in 1 mL of RPMI-1640 medium. On day 3, half of the medium was replaced with fresh medium, and on day 7, cells were harvested for subsequent analysis.

### Single-cell RNA sequencing and data processing

CD45^+^ cells from blood or colorectal tissues were sorted using a BD FACSAria™ III Flow Cytometer Sorter (BD Biosciences). The samples were processed for library preparation using the BD Rhapsody Single Cell Platform (BD Biosciences). Briefly, cells were washed twice with FACS buffer and resuspended in cold Sample Buffer (BD Biosciences, Cat: 650000062). High-quality samples (with > 85% viability as determined by trypan blue staining) were selected for the subsequent procedures. Then, different tissue cell samples were incubated with the hashing sample tag antibodies from the BD™ Human Single-Cell Multiplexing Kit (BD Biosciences, Cat: 633781), resuspended in cold BD Sample Buffer, pooled together (15,000 cells from each sample) to a total of approximately 75,000 cells in 620 µl volume, and loaded into a BD Rhapsody cartridge for a 15 min incubation at 25 °C. Subsequently, Enhanced Cell Capture Beads (BD Biosciences, Cat: 700027881) were loaded onto the cartridge and incubated at 25 °C for 3 min. These cells were then lysed and the released mRNA was captured by Enhanced Cell Capture Beads. The mRNA was then extracted, washed, and subjected to reverse transcription and exonuclease I treatment. cDNA and VDJ libraries were prepared using the BD Rhapsody™ WTA Amplification Kit (Cat: 633801), and the BD Rhapsody TCR Amplification Kit (Cat: 665345). The final library size distribution (quality) was assessed using an Agilent 2200 TapeStation with a high-sensitivity D5000 ScreenTape, and quantification was performed using the Qubit dsDNA HS Assay Kit (Thermo Fisher) with a Qubit fluorometer. WTA libraries were sequenced on an MGISEQ2000 (BGI) platform with a PE100 read length and VDJ libraries with a PE150 read length. FASTQ files containing sequenced data were analyzed using an online Pipeline (Rhapsody™ WTA Analysis Pipeline v1.11.1, http://scm.rhapsodyanalyzer.com) provided by BD.

### Public single-cell RNA-seq data reanalyzing

We re-analyzed publicly available single-cell RNA-sequencing (scRNA-seq) datasets GSE108989 [28] and GSE205506 [29]. The GSE108989 dataset includes T cells derived from peripheral blood, adjacent normal tissue, and tumor tissue of 12 treatment-naïve pMMR colorectal cancer (CRC) patients. From the GSE205506 dataset, 10 dMMR CRC patients who received anti-PD-1 therapy and analyzed tumor-infiltrating T cells collected before and after treatment were included. Data processing was performed using the Seurat package (version 4.0) in R (version 4.3.0). CD8^+^ T cells were identified based on co-expression of CD3E and CD8A, and clustering of these cells was conducted using the t-distributed stochastic neighbor embedding (*t*-SNE) algorithm.

To characterize the functional states of CD103^−^ or CD103^+^ CD8^+^ T cells, we calculated module scores for specific gene signatures using the “AddModuleScore” function. The stemness signature included genes such as *BCL6, IL7R, CCR7, TCF7, BACH2, CXCR5, and SELL*, while effector function was assessed using a gene set comprising *IL2, TNF, IFNG, FASLG, GZMA, GZMB, GZMK, PRF1, CSF1, CCL3, CCL4, CCL5*, and *GNLY*. The exhaustion signature score was calculated based on the expression of *HAVCR2*, *CTLA4*, *LAG3*, *ENTPD1*, *TOX*, *PRDM1*, *IL2RB*, *CD101*, and *ID2*, while the transcription factor signature score was derived from the expression of *TBX21*, *SATB1*, *IRF4*, *JUN*, *NR4A2*, *EOMES*, *BATF*, and *RORC*. TCR diversity was analyzed and visualized using the “scRepertoire” package (version 1.3.1) in R, including Sankey diagrams for clonal transition and statistical box plots displaying Shannon and inv.Simpson indices. TCR distribution was presented using chord diagrams generated in Microsoft Power BI.

### Statistics

Statistical analyses were performed using GraphPad Prism 8.0 (GraphPad Software, CA, USA), and single-cell RNA sequencing data were analyzed using a R package Seurat in R (version 4.3.0). A normal distribution test of data was performed in GraphPad Prism 8.0 to assess the distribution of the data. Normally distributed data were analyzed using paired Student’s t-test or unpaired Student’s *t*-test. For non-normally distributed variables, the Mann–Whitney *U*-test was performed. Statistical significance was set at *P* < 0.05.

## Results

### CD103⁻CD8⁺ T cells increase in pMMR CRC tumors.

**Fig. 1:**
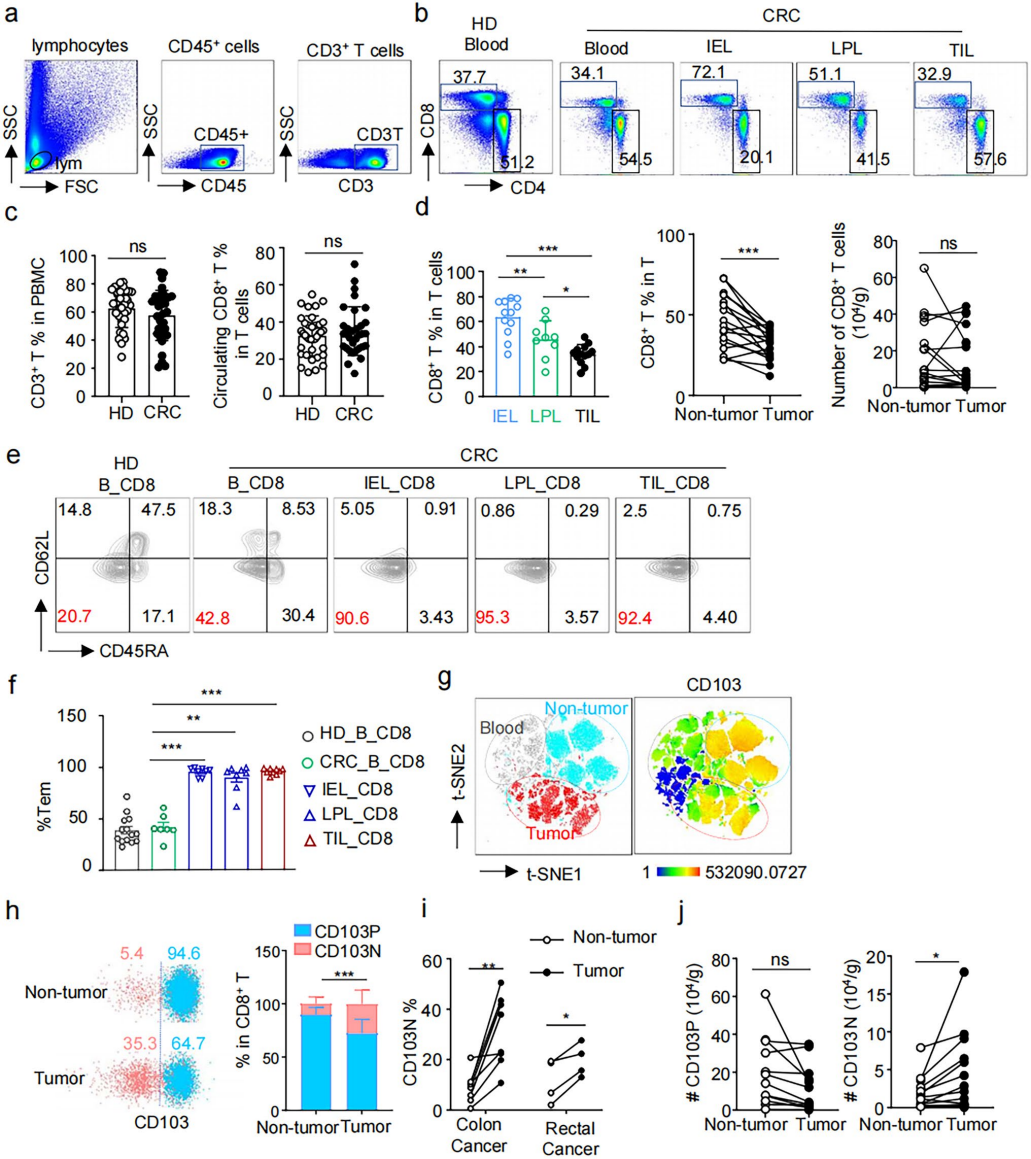
CD103⁻CD8⁺ T cells are increased in pMMR CRC tissues compared to adjacent non-tumor tissues. **a**–**b** Representative flow cytometry plots depicting the gating strategy for identifying CD4⁺ and CD8⁺ T cells within CD3⁺ T cells in peripheral blood of healthy donors (HD) and pMMR CRC patients (CRC), intraepithelial lymphocytes (IEL), lamina propria lymphocytes (LPL), and tumor infiltrating lymphocytes (TIL) of the patients. **c** Bar graphs with scatter plots show the proportion of CD3⁺ T cells among PBMCs from healthy donors (HD, *n* = 37) and CRC (*n* = 34) patients (left), and the proportion of circulating CD8⁺ T cells among CD3⁺ T cells from HD (*n* = 37) and CRC (*n* = 32) groups (right). **d** Bar plots with scatter plots illustrate the proportions of CD8⁺ T cells within infiltrating CD3⁺ T cells across IEL (*n* = 12), LPL (*n* = 9), and TIL (*n* = 13) from CRC patients (left). The middle panel shows the proportion of CD8⁺ T cells among total T cells in non-tumor (*n* = 27) and tumor tissues (*n* = 27), and the right panel shows a comparison of the absolute counts (10^4^/g) of CD8⁺ T cells in tumor (*n* = 15) versus adjacent non-tumor (*n* = 15) tissues. **e** Representative flow cytometry plots exhibit CD45RA and CD62L expression on CD8⁺ T cells from different tissues. **f** A bar graph with scatter plots indicates the proportion of CD45RA⁻CD62L⁻ effector memory T cells (Tem) among CD8⁺ T cells from different tissue compartments. **g** t-SNE analysis of merged CD8⁺ T cells from various tissue origins in CRC patients, performed using FlowJo_V10. The left plot shows the distribution of infiltrating CD8⁺ T cells in non-tumor versus tumor tissues, the right plot displays CD103 expression among these cells. **h** Representative flow cytometry plots (left) and summary bar graphs (right) showing the proportions of CD103⁻ (CD103N) and CD103⁺ (CD103P) cells among CD8⁺ T cells in non-tumor and tumor tissues, *n* = 12. **i** Paired dot plots show the proportion of CD103N cells among CD8⁺ T cells in matched tumor and non-tumor tissues from colon (*n* = 8) and rectal cancer (*n* = 4) respectively. **j** Line graphs quantifying the absolute numbers (10^4^/g) of CD103P (left) and CD103N (right) cells in matched tumor and adjacent non-tumor tissues, *n* = 12

Initially, we analyzed T cells in peripheral blood, non-tumor tissue, and tumor tissue samples from patients diagnosed with proficient mismatch repair (pMMR) colorectal cancer (CRC) (Fig. 1a-b). There was no significant difference in the frequencies of circulating T cells and CD8⁺ T cells between patients and healthy donors (Fig. 1c). In intestine tissue, the ratio of CD8^+^ T cells was highest in intraepithelial lymphocytes (IEL) and lower in lamina propria lymphocytes (LPL) within non-tumor tissues, decreasing to its lowest level in tumor tissue (Fig. 1d, left panel). Consequently, the CD8⁺ T cell ratio in tumor tissue was significantly reduced compared to non-tumor tissue (Fig. 1d, middle panel). In contrast, the number of CD8⁺ T cells per gram of tissue did not differ between tumor and non-tumor tissues (Fig. 1d, right panel), suggesting CD8⁺ T cells infiltration was present in pMMR CRC.

Upon further analysis of CD45RA and CD62L expression on CD8⁺ T cells from different tissues of CRC patients, we found that the majority of CD8⁺ T cells in both non-tumor (IEL and LPL) and tumor tissues predominantly displayed a CD45RA⁻CD62L⁻ effector memory T cell (Tem) phenotype, while those in PBMCs had higher proportions of CD45RA^+^ populations (Fig. 1e-f). Using *t*-SNE dimensionality reduction of markers detected via multicolor flow cytometry, encompassing transcript factors and cell phenotype indicators (Supplementary Table 2), we found that CD8^+^ T cells from the peripheral blood of healthy individuals, as well as from the peripheral blood, para-tumoral IEL and LPL, and tumor tissues of patients, were distinctly separated (Fig. 1g, left). Marked CD103 expression were observed in CD8⁺ T cells from non-tumor and tumor tissues (Fig. 1g, right), although there was a significant reduction in CD103 expression on CD8⁺ T cells in tumor tissues compared to non-tumor tissues (Fig. 1h). Concordantly, tumor tissues showed increased proportions of CD103⁻CD8⁺ T (CD103N) cells in both colon and rectal cancers (Fig. 1i). We also observed a decrease trend in CD103⁺CD8⁺ T (CD103P) cell numbers in tumor tissues, alongside a significant increase in CD103N cell infiltration (Fig. 1j).

### CD103⁻CD8⁺ T cells exhibit reduced levels of exhaustion markers compared to CD103^+^CD8⁺ counterparts and maintained anti-tumor potential in pMMR CRC.

**Fig. 2:**
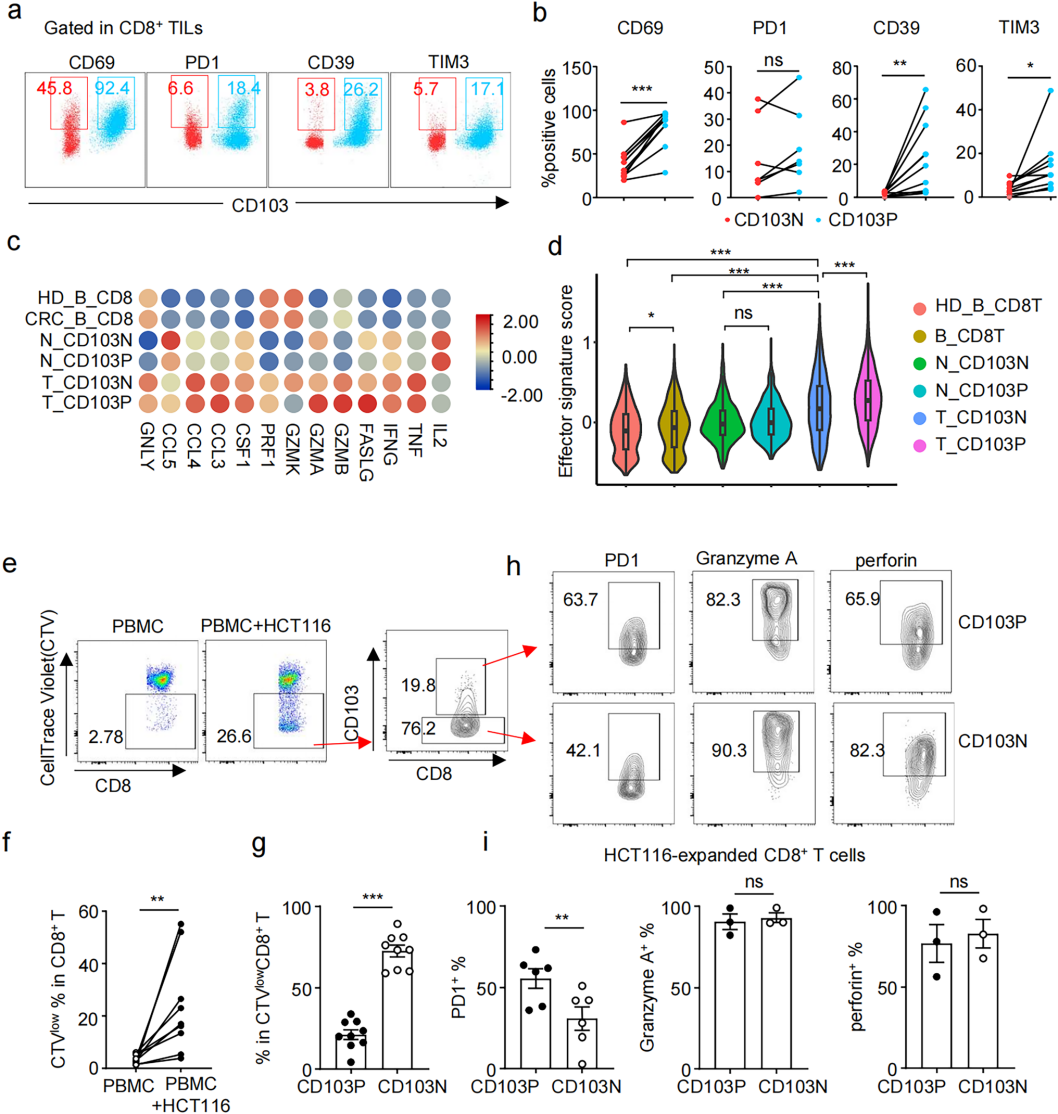
CD103⁻CD8⁺ T cells exhibit lower levels of exhaustion markers and maintained anti-tumor potential in pMMR CRC. **a** Representative flow cytometry plots depict the gating strategy and levels for CD69, PD-1, CD39, and TIM3 expression in CD103N and CD103P cells from tumor tissues. **b** Line plots show the proportions of CD103N and CD103P cells expressing the indicated activation and exhaustion markers in tumor tissues. Each dot represents an individual sample. CD69 (*n* = 10), PD-1 (*n* = 7), CD39 (*n* = 10), TIM3 (*n* = 10). **c** A heatmap shows the expression levels of indicated genes in CD103N and CD103P cells across different tissues based on our own single-cell RNA-sequencing data. **d** Violin plots display effector gene scores for CD103N and CD103P cells across the indicated groups. **e** Representative flow cytometry plots and show the proportion of proliferated CD8⁺ T with reduced CellTrace Violet levels (CTV^low^) in total CD8^+^ T cells in the PBMC only (left panel) and PBMC + HCT116 co-culture (mid panel) groups. Representative flow cytometry plot shows the proportions of CD103P and CD103N cells in proliferated CD8⁺ T cells (right panel). **f** A paired line plot shows the proportion of CTV^low^CD8⁺ T cells in the PBMC-only and the PBMC + HCT116 co-culture groups, *n* = 9. **g** A bar graph with scatter plots displays the proportions of CD103P and CD103N cells among the proliferated CD8⁺ T cells following HCT116 stimulation **h**–**i** Representative flow cytometry plots (top) and summary bar graphs (bottom) show the expression levels of PD-1, Granzyme A, and perforin in expanded CD103N and CD103P cells in indicated groups

Intratumoral CD8⁺ T cells showed reduced CD69 activation marker expression but upregulated exhaustion markers PD-1 and TIM-3 compared to non-tumor tissues, while CD39 expression remained comparable between both sites (supplementary Fig. 1a-b). Notably, CD103N cells displayed lower levels of CD69, CD39, and TIM-3 compared to CD103P cells, and their PD-1 levels were lower in CD103N in 5 out of 7 samples (Fig. 2a-b). Increased CD103N cell with lower activation and exhaustion levels was supposed to be responsible for the reduced CD69 levels and comparable CD39 levels in intratumoral CD8⁺ T cells in pMMR CRC as compared to non-tumor tissue. Additionally, CD8^+^ T cells from both tumor and non-tumor tissues demonstrated comparable capacities to produce anti-tumor cytokines upon PMA and ionomycin stimulation, including IFN-*γ*, TNF-*α*, and IL-2 (supplementary Fig. 1c-d), despite the higher proportion of CD103N cells in the tumor tissue.

To investigate the phenotypic and functional differences between CD103N and CD103P T cells in tumor tissue, we analyzed single-cell RNA sequencing data from our group alongside the publicly available dataset GSE108989 [28]. Based on the levels of *ITGAE*, the gene encoding CD103, CD8^+^ T cells from both datasets were classified into CD103P and CD103N subsets. The anti-tumor capacities of both populations were assessed by comparing the transcript levels of effector molecules, including those encoding for anti-tumor cytokines, cytotoxic mediators, and chemokine ligands that facilitate T cell migration. Both CD103P cells (T_CD103P) and CD103N cells (T_CD103N) infiltrating in tumor tissues exhibited increased expression of anti-tumor genes, including those encoding for cytokines (*TNF, IFNG*), cytotoxic molecules (*GZMB, GZMK, PRF1, GNLY, FASLG, LAMP3*), and chemokine ligands, compared to their counterparts in peripheral blood (CRC_B_CD8) and non-tumor tissues (N_CD103P and N_CD103N) (Fig. 2c). As a result, tumor-infiltrating T_CD103P and T_CD103N cells demonstrated significantly elevated anti-tumor effector signatures score than their counterparts in non-tumor tissues, albeit T_CD103P displayed higher score compared to T_CD103N cells (Fig. 2d).

To further validate the phenotypic and functional differences between CD103N and CD103P cells observed in patients, we set up in vitro stimulation of CD103N and CD103P cells from PBMCs with HCT116 tumor cells. HCT116 cells stimulation significantly enhanced CD8⁺ T cell proliferation, as reflected by increased CellTrace Violet (CTV) dilution in CD8⁺ T cells from PBMC + HCT116 group versus PBMC controls (supplementary Fig. 2a, Fig. 2e-f). Notably, HCT116-expanded CD8^+^ T cells included more CD103N cells than CD103P cells, indicating the better proliferative responses of CD103N cells to HCT116 stimulation (Fig. 2g). In addition, HCT116-expanded CD103N cells exhibited lower levels of PD-1 but comparable levels of cytotoxic mediators including granzyme A and perforin versus CD103P counterparts (Fig. 2h-i).

### CD103⁻CD8⁺ T cells in pMMR CRC exhibit enhanced stem-like TPEX phenotype

**Fig. 3:**
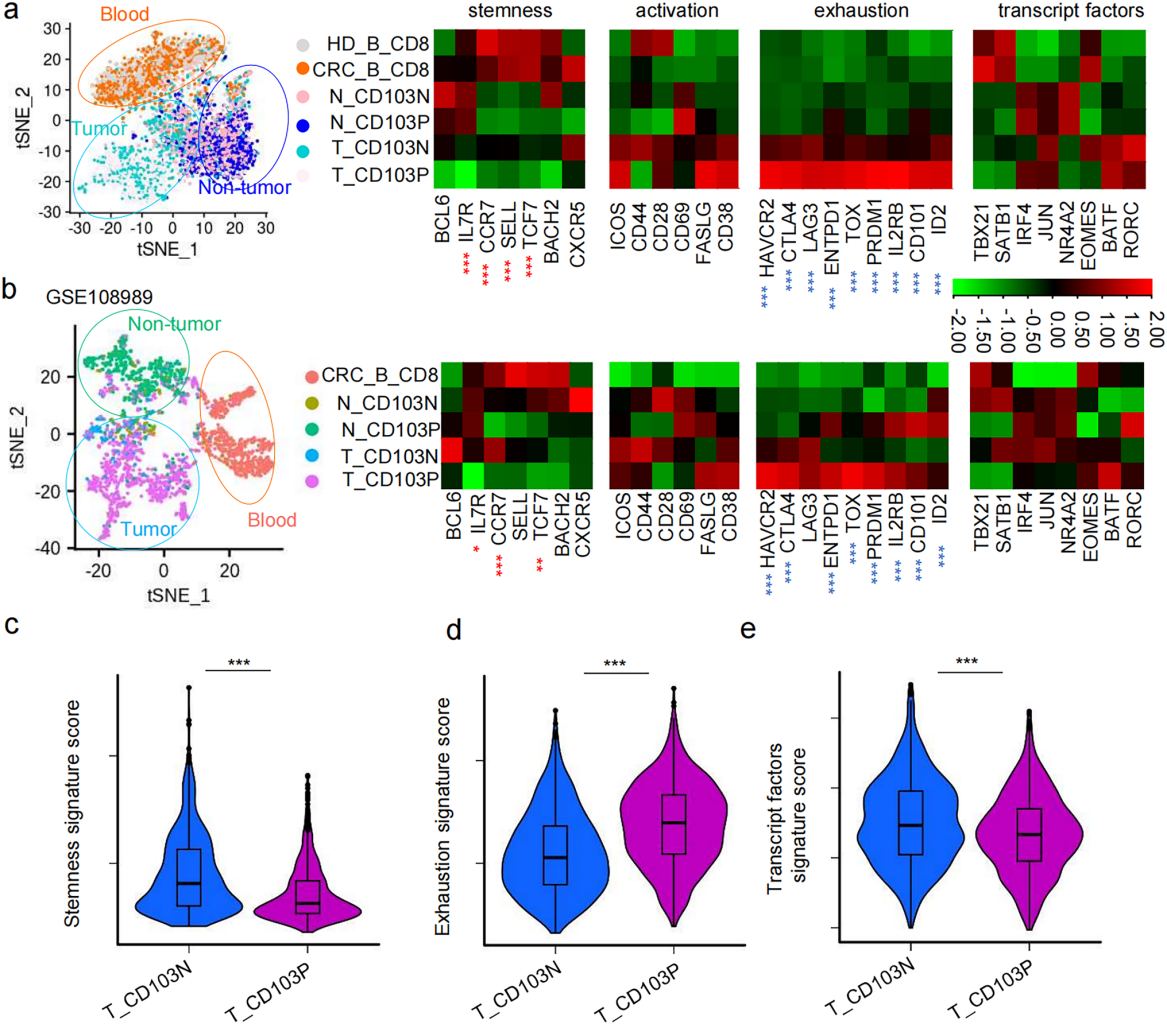
CD103⁻CD8⁺ T cells in pMMR CRC exhibit enhanced stem-like T_PEX_ phenotype. **a**–**b** The t-SNE dimensionality reduction plots with seurat_clusters based on single-cell transcriptomic signatures of peripheral blood of HD (HD_B_CD8), and peripheral blood (CRC_B_CD8), non-tumor tissues (N_CD103N and N_CD103P), and tumor tissues (T_CD103N and T_CD103P) from 1 pMMR CRC patients from our own dataset (a, left) and 12 pMMR CRC patients from public data GSE108989 (b, left), and the heatmaps display the indicated gene expression levels corresponding in indicated cell groups. The statistical significance markers denote the comparison of corresponding genes in T_CD103N versus T_CD103P cells, with red stars indicating upregulated genes while blue stars indicating downregulated genes. (**c**-e) Violin plots display gene signature scores of stemness **c**, exhaustion **d**, and transcription factor **e** for CD103N and CD103P cells in pMMR CRC tissues, calculated using the "AddModuleScore" function on our single-cell RNA data

t-SNE dimensionality analysis revealed that CD8^+^ T cells from the peripheral blood of pMMR CRC patients and healthy individuals clustered together, topographically distinct from CD8^+^ T cells originating from non-tumor colon tissues and tumor tissues in our dataset (Fig. 3a, left) and public dataset GSE108989 (Fig. 3b, left). The transcript levels of markers associate with T cell stemness, activation, exhaustion, and transcript factors in CD8⁺ T cells from patients’ peripheral blood (CRC_B_CD8) were comparable to those in healthy individuals (HD_B_CD8) (Fig. 3a, right), indicating minimal phenotypic changes in circulating CD8⁺ T cells between patient and HD. Notably, CD103N and CD103P cells from non-tumor and tumor tissues exhibited lower transcript levels of stemness markers, including *CCR7, SELL, and TCF7,* compared to circulating CD8^+^ T cells (Fig. 3a-b), resulting in lower stemness signature scores (Fig. 3c).

When comparing T_CD103P cells in tumor tissues to N_CD103P in non-tumor tissues, we found that T_CD103P exhibited higher levels of exhaustion markers but lower levels of stemness signatures (Fig. 3a-b). In contrast, T_CD103N cells tissue consistently exhibited higher levels of stemness markers (*IL7R, CCR7, SELL, TCF7*) and lower levels of exhaustion markers (*HAVCR2, CTLA4, LAG3, ENTPD1, TOX, PRDM1, IL2RB, CD101, ID2*) compared to T_CD103P cells (Fig. 3a-b). The upregulated gene profiles in T_CD103P cells were strongly associated with tissue-resident memory T cells (T_RM_) and T_EX_ cell. In contrast, the upregulated genes in T_CD103N cells aligned more closely with characteristics of T_PEX_ cells, showing lower exhaustion levels but higher levels of stemness score and transcript factors linked to effector mediators than T_CD103P (Fig. 3c-e).

### CD103N cells and CD103P cells share TCR clonotypes, with the same clones distributing across different tissues in pMMR CRC patients.

**Fig. 4:**
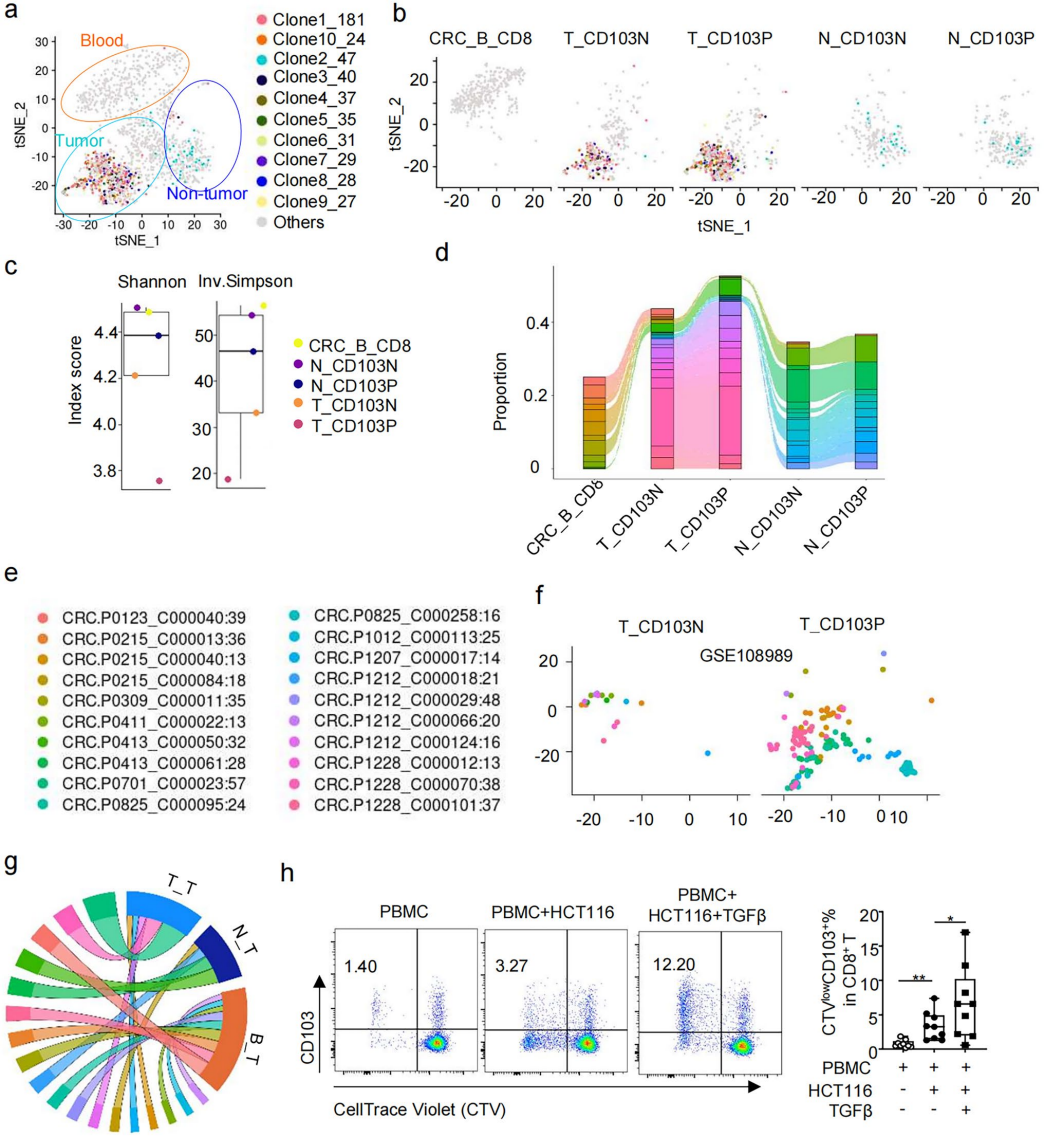
CD103N cells and CD103P cells in tumor tissue share TCR clonotypes. (**a**–**d**) TCR clonotypes distribution of CD103N and CD103P across different tissues from our dataset. **a** t-SNE seurat_clusters plot with the top 10 ranked TCR clonotypes highlighted in distinct colors and other clonotypes in gray. **b** t-SNE seurat_clusters plots stratified by tissue origin and CD103N/CD103P subsets. **c** Shannon and inverse Simpson diversity indices were calculated for CD8^+^ T cells in different groups, with statistical plots showing the respective scores across groups. **d** A Sankey diagram depicts the distribution of CD8^+^ T cells with identical TCR clonotypes among CD103N and CD103P cells across different tissues, each color represents a distinct TCR clonotype. (**e**–**g**) TCR clonotypes distribution of CD103N and CD103P across different tissues from GSE108989 dataset. **e** List of top 20 TCR clonotypes identified. **f** Distribution of the top 20 TCR clonotypes in CD103N and CD103P cells within tumor tissues. **g** A chord diagram illustrates the distribution of CD8^+^ T cells with the top 20 most abundant TCR clonotypes across different tissues, with “B_T” indicating clonotypes shared between blood and tumor tissues, “N_T” indicating clonotypes shared between non-tumor tissues and tumor tissues, and “T_T” representing clonotypes exclusive to tumor tissues. **h** Peripheral blood mononuclear cells (PBMCs) from healthy donors (*n* = 9) were co-cultured with HCT116 cells for 7 days with or without TGF-*β*. Representative flow cytometry plots and box plot show the proportion of CTV^low^CD103⁺CD8⁺ T cells in PBMC only group, PBMC + HCT116 co-culture group, and TGF-*β*-treated group

To evaluate clonal transitions between CD103N and CD103P cells across blood, non-tumor tissue and tumor tissue in CRC, we analyzed the distribution of the most enriched T cell clones in different tissues from pMMR CRC patient via TCR repertoire matching. In our dataset, 4,520 CD8^+^ T cells were sourced from the peripheral blood, non-tumor tissues and tumor tissues of patients, and 1,705 cells were detected to have matching TCR *α*-*β* chains or *γ*-δ chains, encompassing 804 different TCR clonotypes. Among these, 151 TCR clonotypes were represented by two or more cells, with the enriched clones labeled in descending order of clonal frequency (Supplementary Table 3). Among the top ten enriched clones, nine were predominantly found in tumor tissues, with only one clone (Clone2_47) primarily accumulating in non-tumor tissues (Fig. 4a-b). These findings aligned with the clonal expansion of tumor-reactive CD8^+^ T cells within TME. As assessed by Shannon and inverse Simpson indices, the lowest TCR diversity was observed in T_CD103P cells, followed closely by T_CD103N cells, then N_CD103P, N_CD103N and CRC_B_CD8 (Fig. 4c).

Of note, a high overlap rate was observed between CD103N and CD103P clones in both non-tumor and tumor tissues, and shared clones were observed across the patient's peripheral blood, non-tumor tissue, and tumor tissue (Fig. 4d). In the GSE108989 dataset, there were 2,186 CD8^+^ T cells with match TCR *α*-*β* chains and 1,170 distinct TCR clonotypes sourced from peripheral blood, non-tumor tissues, and tumor tissues (Supplementary Table 3). Among the 395 cells from the top enriched 20 clonotypes (Fig. 4e), we also observed the clonotype overlap between CD103N and CD103P cells in the tumor tissue (Fig. 4f), with frequently observed clonotypes distributed across blood, non-tumor tissue and/or tumor tissue (Fig. 4g). These findings provide evidence for the clonotype transition between CD103N and CD103P cells, and suggest that these shared TCR clones are distributed across different tissues in pMMR CRC patients. Consistently, CD8⁺ T cells within PBMCs proliferated and upregulated CD103 expression upon HCT116 cell stimulation. Notably, in the presence of TGF-*β*, the proportion of proliferative CD103⁺CTVlow CD8⁺ T cells was further elevated (Fig. 4h).

### CD103⁻CD8⁺ T cell-enriched subpopulations in pMMR CRC exhibit elevated stem-like TPEX phenotype.

**Fig. 5:**
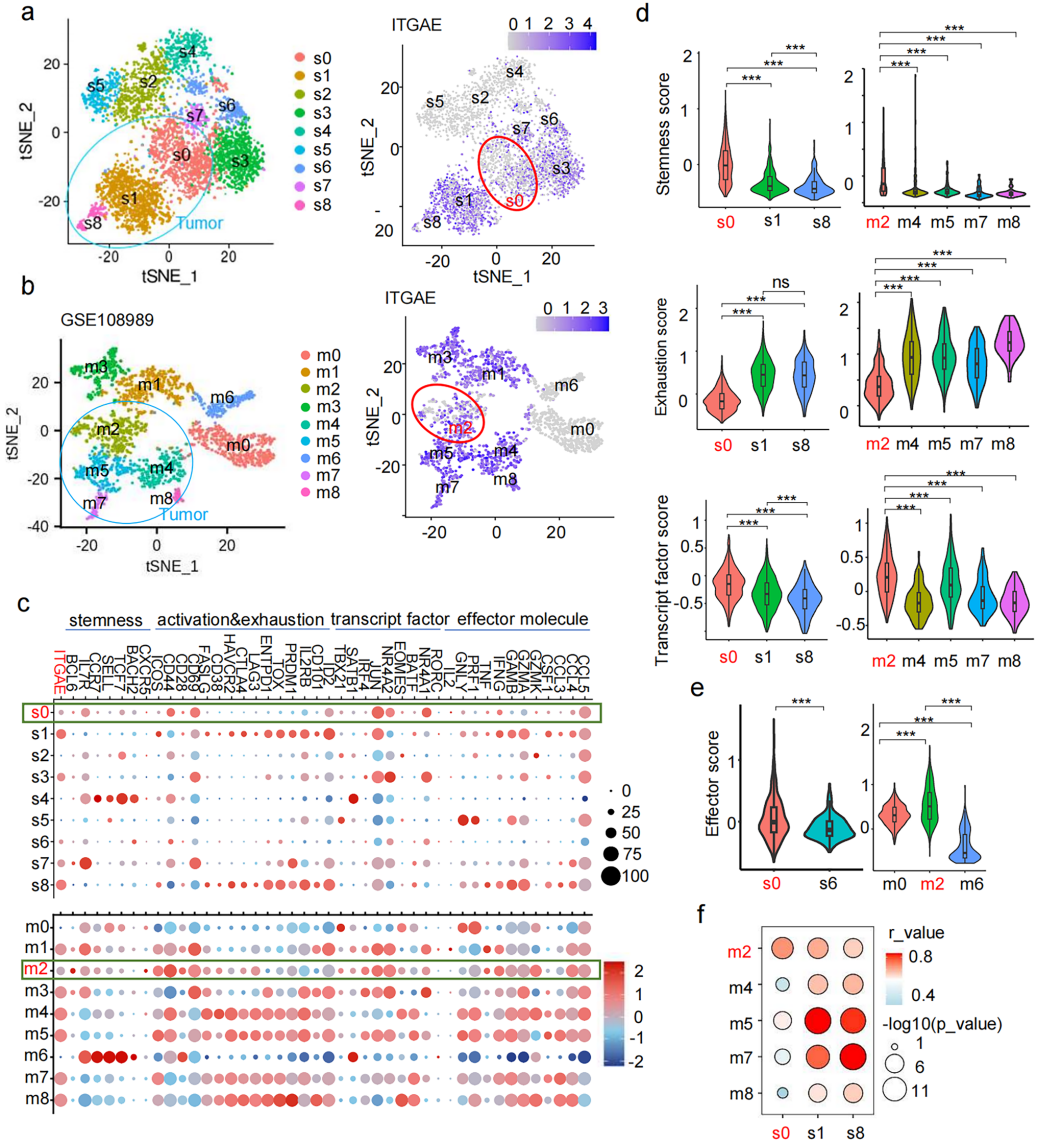
CD103⁻CD8⁺ T cell-enriched subpopulations in pMMR CRC exhibit stem-like T_PEX_ phenotype. **a**–**b** t-SNE seurat_clusters plot shows the 9 unsupervised clusters of CD8⁺ T cells identified in our dataset (s0–s8, a) and GSE108989 dataset (m0-m8, b) with clusters enriched in tumor-infiltrating CD8⁺ T cells highlighted in blue circles (left), and clusters with lower *ITGAE* expression highlighted in red circles (right). **c** Dot plots display the proportion and expression level of the indicated genes across CD8⁺ T cell subsets. **d** Violin plots show the scores for stemness, exhaustion, and transcription factor gene signatures in corresponding CD8⁺ T cell subsets. **e** Violin plots show the effector gene signature scores of CD8⁺ T cell subsets. **f** Dot plot illustrates transcriptomic similarity across different clusters identified between our dataset and the GSE108989 dataset

We further subdivided the CD8^+^ T cells from CRC patients into nine subpopulations based distinct signature genes in tSNE dimensionality reduction analysis (Supplementary Table 4). Of note, tumor-infiltrating CD8^+^ T cells from our dataset were predominantly clustered in populations s0, s1, and s8 (Fig. 5a, left), whereas those from the GSE108989 dataset were clustered in populations m2, m4, m5, m7, and m8 (Fig. 5b, left). CD103N cells in tumor tissue, characterized by low *ITGAE* levels, were primarily clustered in populations s0 and m2, albeit s0 and m2 populations also contain some CD103P cells. However, tumor-infiltrating CD103P cells were clustered across populations s1, s8, m4, m5, m7 and m8 (Fig. 5a-b, right). This suggested that CD103N cells in tumor tissue were relatively homogeneity in their overall transcriptomic signatures, while CD103P cells exhibit greater heterogeneity.

Specially, population s0 and m2 consistently demonstrated higher expression levels of stemness markers, such as *IL7R*, *BCL6*, *CXCR5* and *TCF7*, while exhibiting lower levels of exhausted markers including *ENTPD1, Tim3, CTLA4*, etc., which aligns with the phenotype of T_PEX_ cells (Fig. 5c). Moreover, population s0 and m2 had higher stemness signature scores and effect-related transcript factors scores, but lower scores of exhaustion and effector signatures (Fig. 5d). While s0 and m2 did not show higher transcript levels of effector molecules compared to other tumor-enriched populations, they demonstrated elevated effector signatures relative to s6 in non-tumor tissues and m0/m6 in blood, respectively (Fig. 5e). All these populations exhibited similarly low *ITGAE* levels. Similar to the strong positive correlations observed among CD103P populations across both our dataset and the GSE108989 dataset, a strong correlation was also noted between population s0 and population m2 (Fig. 5f). These correlations were evident across multiple transcript signatures, including transcriptomic signature levels of stemness, activation, exhaustion, transcript factors, and effector molecules (Fig. 5f). This indicated that similar CD8^+^ T cells subpopulations with CD103N enrichment and elevated T_PEX_ phenotype are consistently presented in pMMR CRCs.

### CD103⁻CD8⁺ clonotypes that spread across both peripheral blood and tumor tissues display stem-like TPEX phenotype in pMMR CRC tissue.

**Fig. 6:**
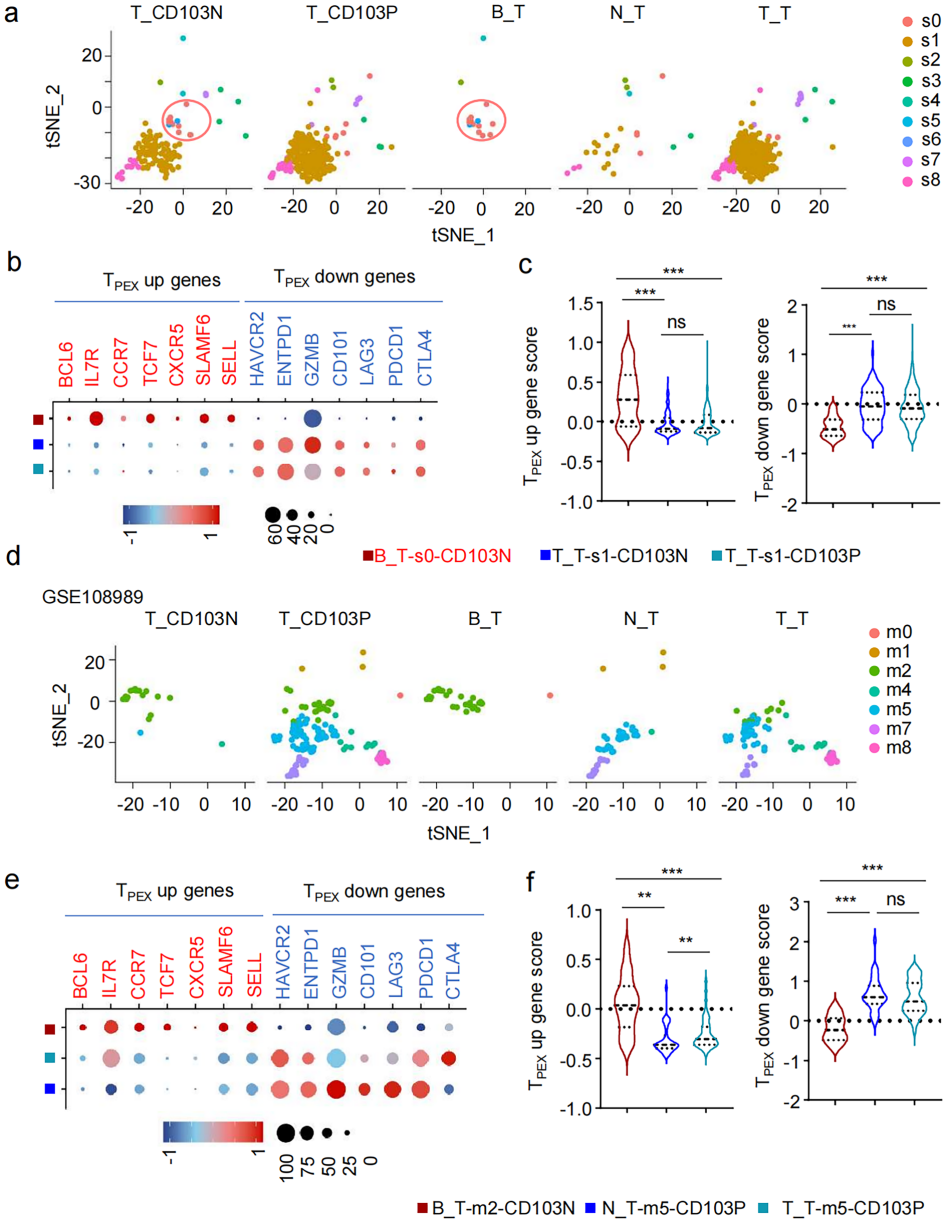
Subpopulations of CD103⁻CD8⁺ T cells across peripheral blood and tumor tissues contained stem-like T_PEX_ clones. (**a** and **d**) t-SNE seurat_clusters plots show the distribution and populations of the top 20 most enriched TCR clonotypes from our dataset **a** and GSE108989 dataset **d** in T_CD103N and T_CD103P, as well as the clones shared between peripheral blood and tumor tissues (B_T), between non-tumor and tumor tissues (N_T), and restricted to tumor tissues (T_T). (**b** and **e**) Bubble plots showing the expression levels of genes up-regulated and down-regulated in T_PEX_ cells in indicated cell groups. *B_T-s0-CD103N* includes the prefix “B_T” designating clonotypes shared in blood and tumor compartments, “s0” representing the cluster, and “CD103N” indicating the cell group. (**c** and **f**) Violin plots depicting the T_PEX_ up-regulated gene scores (left) and down-regulated gene scores (right) across different cell populations

After projecting the top 20 enriched clonotypes from our dataset onto a tSNE dimensionality reduction plot, we observed that shared TCR clones between peripheral blood and tumor tissue (B_T) primally belong to CD103N cells and clustered in population s0 in tumor tissue, which thereafter named as B_T-s0-CD103N. However, clonotypes enriched exclusively in tumor tissue (T_T) were predominantly found in population s1 (Fig. 6a), which contains both CD103N (T_T-s1-CD103N) and CD103P (T_T-s1-CD103P_ clones. Compared to T_T-s1-CD103N and T_T-s1-CD103P, B_T-s0-CD103N cells in tumor tissues exhibited higher levels of stem-like genes characteristic of T_PEX_, including upregulated gene sets associated with stemness, as well as lower levels of gene sets that were downregulated in T_PEX_ cells compared to exhausted T cells. (Fig. 6b-c).

To verify our finding, we performed t-SNE analysis on CD103N cells and CD103P cells and projected the top 20 most enriched TCR clones from the database GES108989. Enriched clonotypes of population m2 containing both CD103N (B_T-m2-CD103N) and CD103P (B_T-m2-CD103P) cells were also clustered across peripheral blood and tumors (Fig. 6d). The B_T-m2-CD103N cells also had higher expression levels of T_PEX_ upregulated genes but lower levels of T_PEX_ downregulated genes, compared to N_T-m5-CD103P cells that distributed across non-tumor tissue and tumor tissue, and T_T-m5-CD103P that were restricted to tumor tissue (Fig. 6e-f). Therefore, both B_T-s0-CD103N from our dataset and B_T-m2-CD103N from public dataset were distributed across both blood and tumor tissue and consistently exhibited T_PEX_ phenotype in CRC tissue. This finding suggests that CD103N T_PEX_ clonotypes replenish the T cell pool of pMMR CRC tissue, which likely arising from blood circulation.

### CD103⁻CD8⁺ clonotypes in tumor tissue demonstrate greater anti-tumor potential compared to their clonally matched peripheral counterparts.

**Fig. 7:**
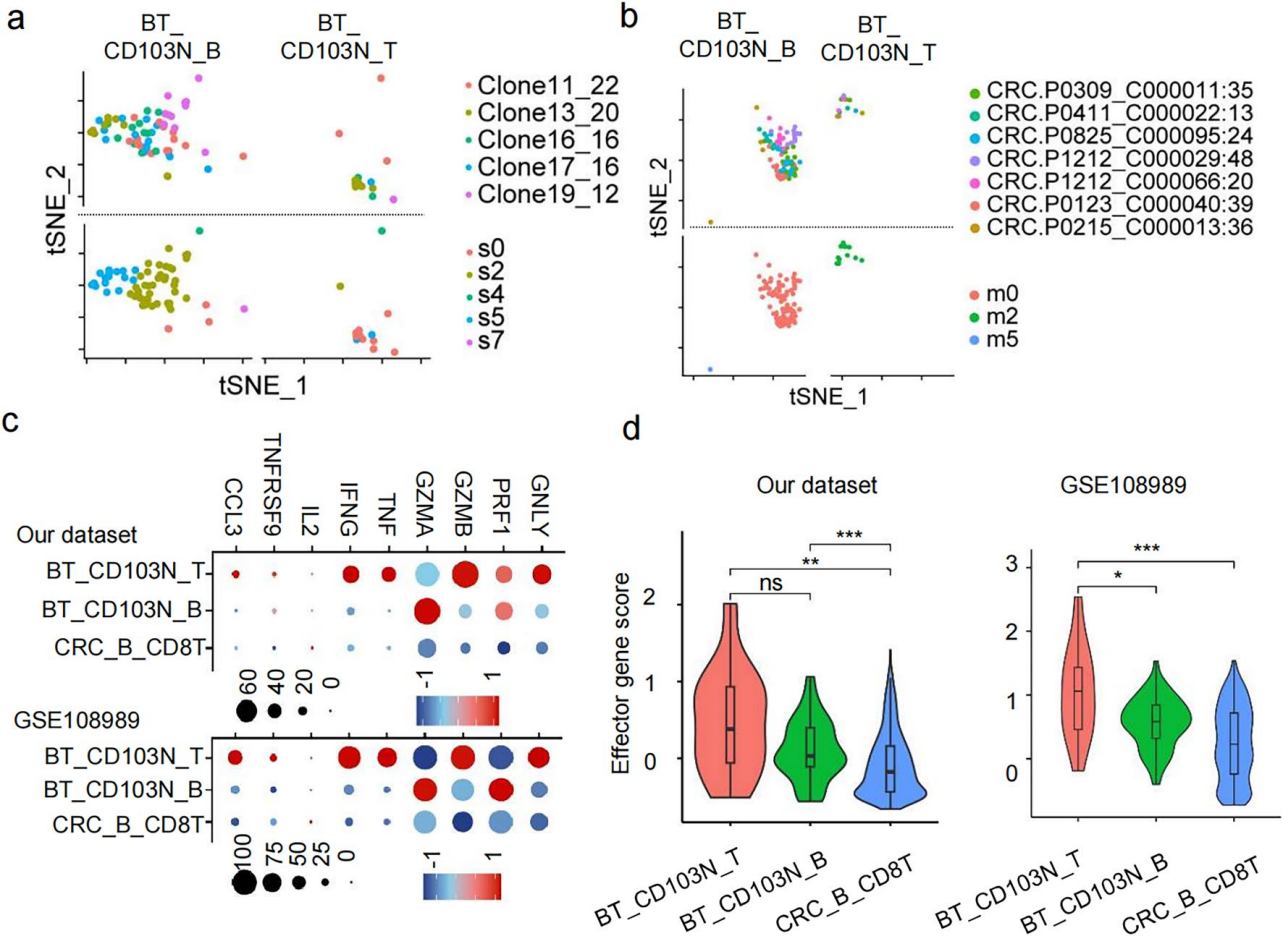
CD103⁻CD8⁺ T cells with shared TCR clonotypes show different levels of anti-tumor potential in tumors and blood. (**a** and **b**) The clonotypes and unsupervised clusters of the top 20 most enriched CD103N clones shared between peripheral blood and tumor tissues (B_T) distributed in blood (BT_CD103N_B) and tumor tissues (BT_CD103N_T) respectively in our data **a** and GSE108989 **b**. **c** Dot plots illustrate the expression frequency and expression levels of selected genes across BT_CD103N_B, BT_CD103N_T, and the total peripheral CD8⁺ T cell population (CRC_B_CD8T). **d** Violin plots compare effector gene scores, calculated based on *IL2, IFNG, TNF, GZMA, GZMB, PRF1* and *GNLY* among indicated cell groups

To evaluate functional differences among CD103N clones with shared TCR clonotypes between peripheral blood and tumor tissues, we isolated CD103N cells from the top 20 most abundant TCR clones (B_T-CD103N) (Fig. 7a-b, upper panel). Among these, peripheral blood-derived cells were designated BT_CD103N_B, while tumor tissue-infiltrating cells were labeled BT_CD103N_T. BT_CD103N_T primally clustered in population s0, matching B_T-s0-CD103N population in tumor tissue mentioned above (Fig. 7a, lower panel). Consistent patterns were observed in the GSE108989 dataset, where BT_CD103N_B and BT_CD103N_T cells were respectively localized in distinct clusters m0 and m2 (Fig. 7b, lower panel). Compared to both BT_CD103N_B and the overall peripheral CD8⁺ T cell population (CRC_B_CD8T), BT_CD103N_T cells exhibited higher expression of *CCL3* and *TNFRSF9*, which are associated with antigen-specific T cell responses, as well as effector mediators linked to anti-tumor potential, including *IFNG*, *TNF*, *GZMB*, *PRF1*, and *GNLY* (Fig. 7c). As compared to BT_CD103N_B, BT_CD103N_T cells in this dataset also exhibited higher expression of *TNFRSF9*, *CCL3*, *IFNG*, *TNF*, *GZMB*, and *GNLY,* with *PRF1* downregulation as the sole exception (Fig. 7c). Consequently, BT_CD103N_T cells from tumor tissue in both datasets displayed higher anti-tumor effector signature scores compared to their circulating counterparts (BT_CD103N_B) and bulk peripheral CD8⁺ T cells (Fig. 7d).

### TPEX-like CD103^−^CD8^+^ T cells are associated with improved response to anti-PD-1 treatment in dMMR CRC patients.

**Fig. 8:**
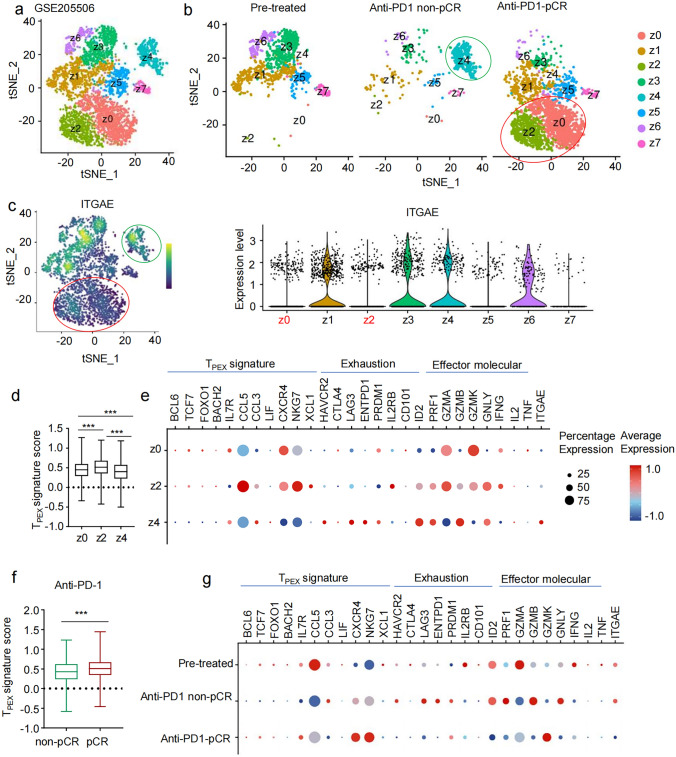
T_PEX_-like CD103^−^CD8^+^ T cells are associated with improved response to anti-PD-1 treatment in dMMR CRC. **a** t-SNE seurat_clusters plot shows the unsupervised clusters of CD8⁺ T cells (z0-z7) from publicly available single-cell RNA-seq dataset GSE205506, including 10 dMMR CRC patients before and after anti-PD-1 treatment. **b** t-SNE seurat_clusters plots stratified by pre-treatment (Pre-treated), non-pathological complete response patients (Anti-PD1 non-pCR) and pathological complete response (Anti-PD1 pCR) post anti-PD-1 treatment. **c** Gene expression density plot (left) and violin plot (right) illustrate levels of *ITGAE* in indicated clusters. **d** T_PEX_ gene signature scores for indicated clusters. **e** Dot plot displays the proportion and expression levels of the indicated T_PEX_ signature, and exhaustion and effector gene sets in indicated clusters. **f** T_PEX_ gene signature scores in CD8⁺ T cells from anti-PD-1 non-pCR and pCR groups. **g** Dot plot shows the expression proportion and levels of the indicated T_PEX_ signature, exhaustion, and effector gene sets across Pre-treated, anti-PD-1 non-pCR, and anti-PD-1 pCR CD8⁺ T cells

To investigate the responsiveness of the stem-like T_PEX_ subpopulation of CD103N cells to anti-PD-1 therapy, we analyzed a publicly available single-cell sequencing dataset (GSE205506). By applying unsupervised clustering, we identified eight distinct populations from z0 to z7 (Fig. 8a). Notably, the different subsets of CD8^+^ T cells exhibited distinct distribution patterns when compared across three groups: patients before anti-PD-1 treatment (pre-treated), those who received anti-PD-1 treatment without pathological remission (anti-PD-1 non-pCR), and those who received anti-PD-1 treatment with pathological remission (anti-PD-1 non-pCR). Specifically, population z4 is enriched in patients of the anti-PD-1 non-pCR group, while populations z0 and z2 are enriched in patients of the anti-PD-1 pCR group (Fig. 8b). Interestingly, we also observed that population z4 highly expressed *ITGAE*, while populations z0 and z2 had low *ITGAE* expression levels (Fig. 8c). Moreover, populations z0 and z2 with low *ITGAE* expression showed higher T_PEX_ upregulated gene score (Fig. 8d). In line with this finding, when compared with population z4, populations z0 and z2 demonstrated elevated expression of T_PEX_ signature genes, including *BCL6, TCF7, FOXO1,* and *BACH2*, as well as effector cell molecules such as *GNLY, PRF1*, and *IFNG*. Conversely, the expression levels of exhaustion-related genes that downregulated in T_PEX_ in populations z0 and z2 were generally lower than those in population z4 (Fig. 8e). Consistent with the increased CD103N cells in pCR group, T cells from pCR patients had higher T_PEX_ signature scores, lower CD103 levels and exhaustion levels, and enhanced cytotoxic functions as compared to the non-pCR group after anti-PD-1 treatment (Fig. 8f-g).

## Discussion

The infiltration level and functional state of CD8^+^ T cells in CRC are critical factors influencing the prognosis and outcomes of the disease [30]. Here, we highlight the enrichment of CD103⁻CD8^+^ T (CD103N) cells in pMMR CRC tumor tissues, which serve as a potential reservoir of T_PEX_ cells with enhanced anti-tumor potential. Furthermore, elevated CD103N cells in dMMR CRC patients correlate with improved response to anti-PD-1 treatment. These CD103N cells, sharing TCR clonotypes across peripheral blood and tumor tissues, present a promising target for recruitment into CRC TME to enhance anti-PD-1therapy.

Unlike in patients with dMMR/MSI-H CRC, immunotherapy alone has not demonstrated a clinical benefit in patients with pMMR/MSS or pMMR/MSI-L CRC, who constitute the vast majority of patients with CRC [31]. The lack of recruitment of immune cells to the tumor seems to be the fundamental obstacle to efficacy [32]. However, our study reveals that pMMR CRC harbors TCR clonotypes identical to those in peripheral blood, predominantly exhibiting a CD103N and T_PEX_ phenotype. Notably, CD103N cells with T_PEX_ characteristics share TCR clonotypes with CD103P cells displaying Tex features. This suggests that a small subset of peripheral blood T cells can migrate into tumor sites, retaining stem-like properties and anti-tumor functionality, while further upregulating CD103 expression within the TME to differentiate into CD103P cells with elevated exhaustion markers and enhanced anti-tumor capacity. Thus, enhancing the chemotaxis and recruitment of CD103N cells to tumor sites while inhibiting their differentiation into Tex cells may represent a novel anti-tumor strategy for pMMR CRC. In dMMR CRC, the increased abundance of CD103N T_PEX_ cells is strongly associated with improved anti-PD-1 treatment responsiveness, providing corroborating evidence for this paradigm.

Extensive research has shown that tissue-resident T cells are abundantly enriched in the intestinal mucosa of both human and mice. Notably, CD103P cells express high levels of the immunomodulatory ectoenzyme CD39, which facilitates the clearance of extracellular ATP under pathological conditions, thereby maintaining intestinal mucosal homeostasis [33]. Moreover, tissue-resident CD8^+^ T cells are a vital and persistent anti-tumor subset within the TME [34]. Studies have further revealed a significant presence of tumor-reactive TCR clonotypes within T_RM_ populations [35], highlighting their critical role in anti-tumor immunity. Our findings revealed that CD103 was highly expressed on CD8^+^ T cells within the epithelial layer of non-tumor intestinal mucosa, with slightly lower expression in the lamina propria. In contrast, we found that the number and proportion of CD103N cells in CRC tumor tissues were significantly higher than those in adjacent normal tissues. Non-resident T cells in the tumor may include a large number of bystander T cells rather than tumor-reactive T cells, which is not conducive to promoting anti-tumor effects. However, it is understood that during the homing and recirculation processes, T cells migrate directionally to the relevant tissues upon encountering antigenic stimulation. This suggests that the increase of CD103N cells in the tumor may be due to the recruitment of tumor microenvironment-reactive T cells from peripheral or other immune organs. Supporting this hypothesis is the finding that both CD103N and CD103P cells in the tumor tissue share a large number of proliferating TCR clones. Moreover, these identical clonotypes of T cells have also been found in the CD103N cells in the patient's peripheral blood. Existing studies demonstrate that CD103P cells cannot convert into CD103N cells, TGF-*β* signaling in the TME drives CD103N cells into CD103P cells [18, 36]. Consistent with this paradigm, we observed that HCT116 cells stimulation significantly increased the proportion of tumor-reactive CD103P cells in the presence of TGF-*β*. It has been reported that TGF-*β* induces Smad3 phosphorylation, which then translocates into the nucleus to promote *ITGAE* transcription, hence upregulating CD103 level. In addition, transcription factors such as Blimp1, Runx3, and Hic1 have been also shown to synergize with TGF-*β* signaling to enhance CD103 expression [37–39]. Moreover, in the intestinal microenvironment, E-cadherin (the ligand for CD103) expressed by intestinal epithelial cells promotes the retention and functional maintenance of CD103⁺ T cells through a "positive feedback" mechanism. This suggests that CD103N cells may be replenished from the patient's peripheral blood to the tumor tissue, and under specific conditions, they can convert into the CD103P T_RM_ phenotype. Therefore, identifying the tumor-reactive subpopulations within CD103N cells is crucial to understanding their significance and importance in tumor therapy.

T_PEX_ cells have been shown to correlate with favorable outcomes in response to anti-PD-1 therapy in various cancers [24, 40]. Our results indicate that CD103N cells in tumor tissues exhibit significantly elevated T_PEX_-related gene expression compared to CD103P cells, while showing slightly lower T_EX_ marker expression. These data suggested that CD103N cells had not fully transitioned to an irreversible terminally exhausted state and might comprise cells at different stages of exhaustion. Consistent with this, single-cell transcriptomic analyses identified two distinct clusters (cluster 0 and cluster 7) within tumor-infiltrating CD103N cells, both exhibiting pronounced T_PEX_ phenotypes. Notably, cluster 0 CD103N cells were found to share identical TCR clonotypes between peripheral blood and tumor tissues. These cells further demonstrated strong upregulation of T_PEX_-related genes and exhibited enhanced production of cytotoxic mediators such as *PRF1* and *GNLY*. These findings supported the existence of a migration pathway through which peripheral blood CD103N cells replenish T_PEX_ cells in tumor tissues. Studies have shown that T_PEX_ cells are enriched in tertiary lymphoid structures or antigen-presenting regions within tumors [41]. Consistent with this, CD103N cells sharing TCR clonotypes between peripheral blood and tumors were found to exhibit high expression of the chemokine receptor CXCR5, which facilitates T cell migration to lymphoid tissues [42]. T_PEX_ cells have been associated with better prognosis in CRC and improved responses to anti-PD-1 therapy [43]. We further found that the T_PEX_-enriched subset of CD103N cells was predominantly abundant in pCR patients rather than in non-pCR patients.

Although we identified this stem-like subset of CD103N cells that may play a role in anti-tumor immunity, we did not investigate their spatial distribution within tumor-draining lymph nodes and tumor tissues. Previous studies have shown that in patients with non-small cell lung cancer and head and neck squamous cell carcinoma, T_PEX_ cells are predominantly located within tertiary lymphoid structures (TLS) and rarely infiltrate the tumor parenchyma. Additionally, in the TME of renal cell carcinoma, stem-like T cells marked by TCF-1 expression tend to cluster in regions densely populated with antigen-presenting cells, which functionally resemble TLS. These findings underscore the importance of such immune niches in shaping the spatial dynamics of T_PEX_ cells within the TME [44]. T_PEX_ cells have been reported to be more enriched in the epithelial regions of tumor tissues in CRC patients [45]. Additionally, we lacked studies exploring the anti-tumor effects of targeting and modulating this subset in murine tumor models. We anticipate that future research will delve deeper into these aspects. In summary, we found that the increased infiltration of CD103N cells in tumor tissues serves as an important reservoir of T_PEX_ cells and is closely associated with the prognosis of patients undergoing anti-PD-1 therapy.

## Supplementary Information

Below is the link to the electronic supplementary material.

Supplementary file1 (DOCX 828 KB)

Supplementary file2 (XLSX 11 KB)

Supplementary file3 (XLSX 10 KB)

Supplementary file4 (XLS 2010 KB)

Supplementary file5 (XLS 67 KB)

## Data Availability

The single-cell RNA-seq data will be made available on Gene Expression Omnibus. The data and analysis code used in the manuscript are available upon reasonable request to the authors.

## References

[1] Xie YH, Chen YX, Fang JY (2020) Comprehensive review of targeted therapy for colorectal cancer. Signal Transduct Target Ther 5(1):22 32296018 10.1038/s41392-020-0116-z PMC7082344

[2] Lu B, Li N, Luo CY, Cai J, Lu M, Zhang YH, Chen HD, Dai M (2021) Colorectal cancer incidence and mortality: the current status, temporal trends and their attributable risk factors in 60 countries in 2000–2019. Chin Med J (Engl) 134(16):1941–1951 34238851 10.1097/CM9.0000000000001619 PMC8382382

[3] Zhou J, Ji Q, Li Q (2021) Resistance to anti-EGFR therapies in metastatic colorectal cancer: underlying mechanisms and reversal strategies. J Exp Clin Cancer Res 40(1):328 34663410 10.1186/s13046-021-02130-2 PMC8522158

[4] Zhou C, Cheng X, Tu S (2021) Current status and future perspective of immune checkpoint inhibitors in colorectal cancer. Cancer Lett 521:119–129 34464671 10.1016/j.canlet.2021.07.023

[5] Boland CR, Goel A (2010) Microsatellite Instability in Colorectal Cancer. Gastroenterology 138(6):2073-U87 20420947 10.1053/j.gastro.2009.12.064 PMC3037515

[6] Markowitz SD, Bertagnolli MM (2009) Molecular origins of cancer: molecular basis of colorectal cancer. N Engl J Med 361(25):2449–2460 20018966 10.1056/NEJMra0804588 PMC2843693

[7] Ganesh K, Stadler ZK, Cercek A, Mendelsohn RB, Shia J, Segal NH, Diaz LA (2019) Immunotherapy in colorectal cancer: rationale, challenges and potential. Nat Rev Gastroenterol Hepatol 16(6):361–375 30886395 10.1038/s41575-019-0126-x PMC7295073

[8] Wala J, De Bruijn I, Coy S, Gagne A, Chan S, Chen Y A, Hoffer J, Muhlich J, Schultz N, Santagata S, Sorger P K (2024) Integrating spatial profiles and cancer genomics to identify immune-infiltrated mismatch repair proficient colorectal cancers. bioRxiv.

[9] Kikuchi T, Mimura K, Okayama H, Nakayama Y, Saito K, Yamada L, Endo E, Sakamoto W, Fujita S, Endo H, Saito M, Momma T, Saze Z, Ohki S, Kono K (2019) A subset of patients with MSS/MSI-low-colorectal cancer showed increased CD8(+) TILs together with up-regulated IFN-*γ*. Oncol Lett 18(6):5977–5985 31788072 10.3892/ol.2019.10953 PMC6865144

[10] Chen LJ, Zheng X, Shen YP, Zhu YB, Li Q, Chen J, Xia R, Zhou SM, Wu CP, Zhang XG, Lu BF, Jiang JT (2013) Higher numbers of T-bet(+) intratumoral lymphoid cells correlate with better survival in gastric cancer. Cancer Immunol Immunother 62(3):553–61 23090288 10.1007/s00262-012-1358-6 PMC11028958

[11] Katz SC, Pillarisetty V, Bamboat ZM, Shia J, Hedvat C, Gonen M, Jarnagin W, Fong Y, Blumgart L, D’angelica M, Dematteo RP (2009) T cell infiltrate predicts long-term survival following resection of colorectal cancer liver metastases. Ann Surg Oncol 16(9):2524–30 19568816 10.1245/s10434-009-0585-3

[12] Koh CH, Lee S, Kwak M, Kim BS, Chung Y (2023) CD8 t-cell subsets: heterogeneity, functions, and therapeutic potential. Exp Mol Med 55(11):2287–2299 37907738 10.1038/s12276-023-01105-x PMC10689838

[13] Paap EM, Müller TM, Sommer K, Neurath MF, Zundler S (2020) Total recall: intestinal T(RM) cells in health and disease. Front Immunol 11:623072 33542725 10.3389/fimmu.2020.623072 PMC7851044

[14] Weeden CE, Gayevskiy V, Marceaux C, Batey D, Tan T, Yokote K, Ribera NT, Clatch A, Christo S, Teh CE, Mitchell AJ, Trussart M, Rankin L, Obers A, Mcdonald JA, Sutherland KD, Sharma VJ, Starkey G, D’costa R, Antippa P, Leong T, Steinfort D, Irving L, Swanton C, Gordon CL, Mackay LK, Speed TP, Gray DHD, Asselin-Labat ML (2023) Early immune pressure initiated by tissue-resident memory T cells sculpts tumor evolution in non-small cell lung cancer. Cancer Cell 41(5):837-852.e6 37086716 10.1016/j.ccell.2023.03.019

[15] Ida S, Takahashi H, Kawabata-Iwakawa R, Mito I, Tada H, Chikamatsu K (2021) Tissue-resident memory T cells correlate with the inflammatory tumor microenvironment and improved prognosis in head and neck squamous cell carcinoma. Oral Oncol 122:105508 34507204 10.1016/j.oraloncology.2021.105508

[16] Edwards J, Wilmott JS, Madore J, Gide TN, Quek C, Tasker A, Ferguson A, Chen J, Hewavisenti R, Hersey P, Gebhardt T, Weninger W, Britton WJ, Saw RPM, Thompson JF, Menzies AM, Long GV, Scolyer RA, Palendira U (2018) CD103(+) tumor-resident CD8(+) T cells are associated with improved survival in immunotherapy-naïve melanoma patients and expand significantly during anti-PD-1 treatment. Clin Cancer Res 24(13):3036–3045 29599411 10.1158/1078-0432.CCR-17-2257

[17] Liu S, Wang P, Wang P, Zhao Z, Zhang X, Pan Y, Pan J (2024) Tissue-resident memory CD103+CD8+ T cells in colorectal cancer: its implication as a prognostic and predictive liver metastasis biomarker. Cancer Immunol Immunother 73(9):176 38954030 10.1007/s00262-024-03709-2 PMC11219596

[18] Christo SN, Evrard M, Park SL, Gandolfo LC, Burn TN, Fonseca R, Newman DM, Alexandre YO, Collins N, Zamudio NM, Souza-Fonseca-Guimaraes F, Pellicci DG, Chisanga D, Shi W, Bartholin L, Belz GT, Huntington ND, Lucas A, Lucas M, Mueller SN, Heath WR, Ginhoux F, Speed TP, Carbone FR, Kallies A, Mackay LK (2021) Discrete tissue microenvironments instruct diversity in resident memory T cell function and plasticity. Nat Immunol 22(9):1140–1151 34426691 10.1038/s41590-021-01004-1

[19] Zhao S, Peralta RM, Avina-Ochoa N, Delgoffe GM, Kaech SM (2021) Metabolic regulation of T cells in the tumor microenvironment by nutrient availability and diet. Semin Immunol 52:101485 34462190 10.1016/j.smim.2021.101485 PMC8545851

[20] Kersten K, Hu KH, Combes AJ, Samad B, Harwin T, Ray A, Rao AA, Cai E, Marchuk K, Artichoker J, Courau T, Shi Q, Belk J, Satpathy AT, Krummel MF (2022) Spatiotemporal co-dependency between macrophages and exhausted CD8(+) T cells in cancer. Cancer Cell 40(6):624-638.e9 35623342 10.1016/j.ccell.2022.05.004 PMC9197962

[21] Daniel B, Yost KE, Hsiung S, Sandor K, Xia Y, Qi Y, Hiam-Galvez KJ, Black M, Raposo CJ, Shi Q, Meier SL, Belk JA, Giles JR, Wherry EJ, Chang HY, Egawa T, Satpathy AT (2022) Divergent clonal differentiation trajectories of T cell exhaustion. Nat Immunol 23(11):1614–1627 36289450 10.1038/s41590-022-01337-5 PMC11225711

[22] Lan X, Zebley CC, Youngblood B (2023) Cellular and molecular waypoints along the path of T cell exhaustion. Sci Immunol 8(87):eadg3868 37656775 10.1126/sciimmunol.adg3868 PMC10618911

[23] Zehn D, Thimme R, Lugli E, De Almeida GP, Oxenius A (2022) “Stem-like” precursors are the fount to sustain persistent CD8(+) T cell responses. Nat Immunol 23(6):836–847 35624209 10.1038/s41590-022-01219-w

[24] Brummelman J, Mazza EMC, Alvisi G, Colombo FS, Grilli A, Mikulak J, Mavilio D, Alloisio M, Ferrari F, Lopci E, Novellis P, Veronesi G, Lugli E (2018) High-dimensional single cell analysis identifies stem-like cytotoxic CD8(+) T cells infiltrating human tumors. J Exp Med 215(10):2520–2535 30154266 10.1084/jem.20180684 PMC6170179

[25] Beltra JC, Manne S, Abdel-Hakeem MS, Kurachi M, Giles JR, Chen Z, Casella V, Ngiow SF, Khan O, Huang YJ, Yan P, Nzingha K, Xu W, Amaravadi RK, Xu X, Karakousis GC, Mitchell TC, Schuchter LM, Huang AC, Wherry EJ (2020) Developmental relationships of four exhausted CD8(+) T cell subsets reveals underlying transcriptional and epigenetic landscape control mechanisms. Immunity 52(5):825-841.e8 32396847 10.1016/j.immuni.2020.04.014 PMC8360766

[26] Gebhardt T, Park SL, Parish IA (2023) Stem-like exhausted and memory CD8(+) T cells in cancer. Nat Rev Cancer 23(11):780–798 37821656 10.1038/s41568-023-00615-0

[27] Sun Q, Cai D, Liu D, Zhao X, Li R, Xu W, Xie B, Gou M, Wei K, Li Y, Huang J, Chi X, Wei P, Hao J, Guo X, Pan B, Fu Y, Ni L, Dong C (2023) BCL6 promotes a stem-like CD8(+) T cell program in cancer via antagonizing BLIMP1. Sci Immunol. 8(88):eadh1306 37862431 10.1126/sciimmunol.adh1306

[28] Zhang L, Yu X, Zheng L, Zhang Y, Li Y, Fang Q, Gao R, Kang B, Zhang Q, Huang JY, Konno H, Guo X, Ye Y, Gao S, Wang S, Hu X, Ren X, Shen Z, Ouyang W, Zhang Z (2018) Lineage tracking reveals dynamic relationships of T cells in colorectal cancer. Nature 564(7735):268–272 30479382 10.1038/s41586-018-0694-x

[29] Li J, Wu C, Hu H, Qin G, Wu X, Bai F, Zhang J, Cai Y, Huang Y, Wang C, Yang J, Luan Y, Jiang Z, Ling J, Wu Z, Chen Y, Xie Z, Deng Y (2023) Remodeling of the immune and stromal cell compartment by PD-1 blockade in mismatch repair-deficient colorectal cancer. Cancer Cell 41(6):1152-1169.e7 37172580 10.1016/j.ccell.2023.04.011

[30] Thibaudin M, Limagne E, Hampe L, Ballot E, Truntzer C, Ghiringhelli F (2022) Targeting PD-L1 and TIGIT could restore intratumoral CD8 T cell function in human colorectal cancer. Cancer Immunol Immunother 71(10):2549–2563 35292828 10.1007/s00262-022-03182-9 PMC10992601

[31] Takei S, Tanaka Y, Lin YT, Koyama S, Fukuoka S, Hara H, Nakamura Y, Kuboki Y, Kotani D, Kojima T, Bando H, Mishima S, Ueno T, Kojima S, Wakabayashi M, Sakamoto N, Kojima M, Kuwata T, Yoshino T, Nishikawa H, Mano H, Endo I, Shitara K, Kawazoe A (2024) Multiomic molecular characterization of the response to combination immunotherapy in MSS/pMMR metastatic colorectal cancer. J Immunother Cancer. 10.1136/jitc-2023-008210 38336371 10.1136/jitc-2023-008210 PMC10860060

[32] Chen L, Jiang X, Li Y, Zhang Q, Li Q, Zhang X, Zhang M, Yu Q, Gao D (2022) How to overcome tumor resistance to anti-PD-1/PD-L1 therapy by immunotherapy modifying the tumor microenvironment in MSS CRC. Clin Immunol 237:108962 35227870 10.1016/j.clim.2022.108962

[33] Li XY, Moesta AK, Xiao C, Nakamura K, Casey M, Zhang H, Madore J, Lepletier A, Aguilera AR, Sundarrajan A, Jacoberger-Foissac C, Wong C, Dela Cruz T, Welch M, Lerner AG, Spatola BN, Soros VB, Corbin J, Anderson AC, Effern M, Hölzel M, Robson SC, Johnston RL, Waddell N, Smith C, Bald T, Geetha N, Beers C, Teng MWL, Smyth MJ (2019) Targeting CD39 in cancer reveals an extracellular ATP: and inflammasome-driven tumor immunity. Cancer Discov 9(12):1754–1773 31699796 10.1158/2159-8290.CD-19-0541 PMC6891207

[34] Molodtsov A, Turk MJ (2018) Tissue resident CD8 memory T cell responses in cancer and autoimmunity. Front Immunol 9:2810 30555481 10.3389/fimmu.2018.02810 PMC6281983

[35] Lee YJ, Kim JY, Jeon SH, Nam H, Jung JH, Jeon M, Kim ES, Bae SJ, Ahn J, Yoo TK, Sun WY, Ahn SG, Jeong J, Park SH, Park WC, Kim SI, Shin EC (2022) CD39(+) tissue-resident memory CD8(+) T cells with a clonal overlap across compartments mediate antitumor immunity in breast cancer. Sci Immunol 7(74):eabn8390 36026440 10.1126/sciimmunol.abn8390

[36] Zhang N, Bevan MJ (2013) Transforming growth factor-*β* signaling controls the formation and maintenance of gut-resident memory T cells by regulating migration and retention. Immunity 39(4):687–96 24076049 10.1016/j.immuni.2013.08.019 PMC3805703

[37] Qiu Z, Khairallah C, Chu TH, Imperato JN, Lei X, Romanov G, Atakilit A, Puddington L, Sheridan BS (2023) Retinoic acid signaling during priming licenses intestinal CD103+ CD8 TRM cell differentiation. J Exp Med. 10.1084/jem.20210923 36809399 10.1084/jem.20210923 PMC9960115

[38] Dean JW, Helm EY, Fu Z, Xiong L, Sun N, Oliff KN, Muehlbauer M, Avram D, Zhou L (2023) The aryl hydrocarbon receptor cell intrinsically promotes resident memory CD8(+) T cell differentiation and function. Cell Rep 42(1):111963 36640340 10.1016/j.celrep.2022.111963 PMC9940759

[39] Xu W, Bergsbaken T, Edelblum KL (2022) The multifunctional nature of CD103 (*α*E*β*7 integrin) signaling in tissue-resident lymphocytes. Am J Physiol Cell Physiol 323(4):C1161-c1167 36036450 10.1152/ajpcell.00338.2022 PMC9576162

[40] Huang Q, Wu X, Wang Z, Chen X, Wang L, Lu Y, Xiong D, Liu Q, Tian Y, Lin H, Guo J, Wen S, Dong W, Yang X, Yuan Y, Yue Z, Lei S, Wu Q, Ran L, Xie L, Wang Y, Gao L, Tian Q, Zhou X, Sun B, Xu L, Tang Z, Ye L (2022) The primordial differentiation of tumor-specific memory CD8(+) T cells as bona fide responders to PD-1/PD-L1 blockade in draining lymph nodes. Cell 185(22):4049-4066.e25 36208623 10.1016/j.cell.2022.09.020

[41] Jansen CS, Prokhnevska N, Master VA, Sanda MG, Carlisle JW, Bilen MA, Cardenas M, Wilkinson S, Lake R, Sowalsky AG, Valanparambil RM, Hudson WH, Mcguire D, Melnick K, Khan AI, Kim K, Chang YM, Kim A, Filson CP, Alemozaffar M, Osunkoya AO, Mullane P, Ellis C, Akondy R, Im SJ, Kamphorst AO, Reyes A, Liu Y, Kissick H (2019) An intra-tumoral niche maintains and differentiates stem-like CD8 T cells. Nature 576(7787):465–470 31827286 10.1038/s41586-019-1836-5 PMC7108171

[42] Ozga AJ, Chow MT, Lopes ME, Servis RL, Di Pilato M, Dehio P, Lian J, Mempel TR, Luster AD (2022) CXCL10 chemokine regulates heterogeneity of the CD8(+) T cell response and viral set point during chronic infection. Immunity 55(1):82-97.e8 34847356 10.1016/j.immuni.2021.11.002 PMC8755631

[43] Luo Y, Liang H (2023) Single-cell dissection of tumor microenvironmental response and resistance to cancer therapy. Trends Genet 39(10):758–772 37658004 10.1016/j.tig.2023.07.005 PMC10529478

[44] Fang Z, Ding X, Huang H, Jiang H, Jiang J, Zheng X (2024) Revolutionizing tumor immunotherapy: unleashing the power of progenitor exhausted T cells. Cancer Biol Med 21(6):499–512 38825813 10.20892/j.issn.2095-3941.2024.0105 PMC11208905

[45] Huang H, Ge J, Fang Z, Wu S, Jiang H, Lang Y, Chen J, Xiao W, Xu B, Liu Y, Chen L, Zheng X, Jiang J (2024) Precursor exhausted CD8(+)T cells in colorectal cancer tissues associated with patient’s survival and immunotherapy responsiveness. Front Immunol 15:1362140 38510246 10.3389/fimmu.2024.1362140 PMC10950923

