# Supplementary material for: Increased CD103−CD8+ TILs with TPEX phenotype replenish anti-tumor T cell pool in mismatch repair-proficient CRC
Source: Cancer Immunol Immunother. 2025 Nov 3;74(12):358. doi: 10.1007/s00262-025-04214-w (PMC12583254; doi:10.1007/s00262-025-04214-w)
Supplement: Supplementary file 1 — Supplementary file1 (DOCX 828 KB) [file 262_2025_4214_MOESM1_ESM.docx]

**Supplementary figure**


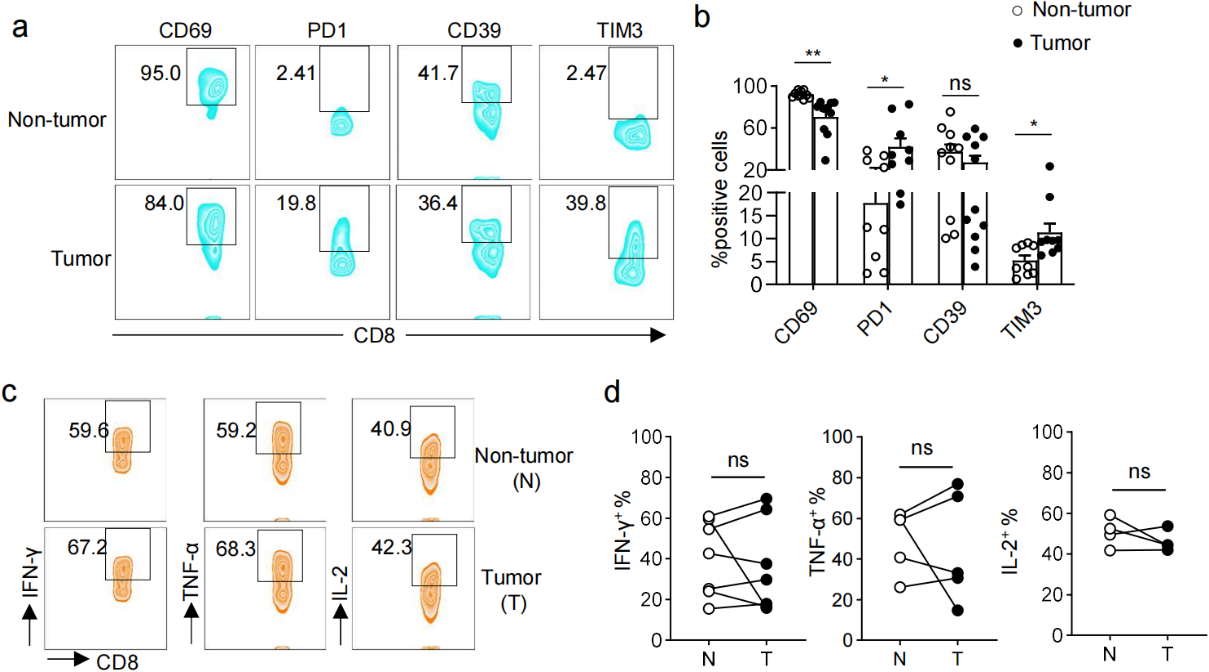


**Supplementary figure 1.** **Phenotypic and functional changes of CD8⁺ T cells in CRC.** (a-b) Representative flow cytometry plots (a) and statistical plots (b) showing the proportions of CD69 (n=10), PD-1 (n=9), CD39 (n=10), and TIM3 (n=9) expression in CD8^+^ T cells from non-tumor tissues and tumor tissues. (e-f) Representative flow cytometry plots (e) and summary bar graphs (f) display the proportions of IFN-γ- (n=7), TNF-α- (n=5), and IL-2- (n=4) producing CD8^+^ T cells from non-tumor and tumor tissues.

**
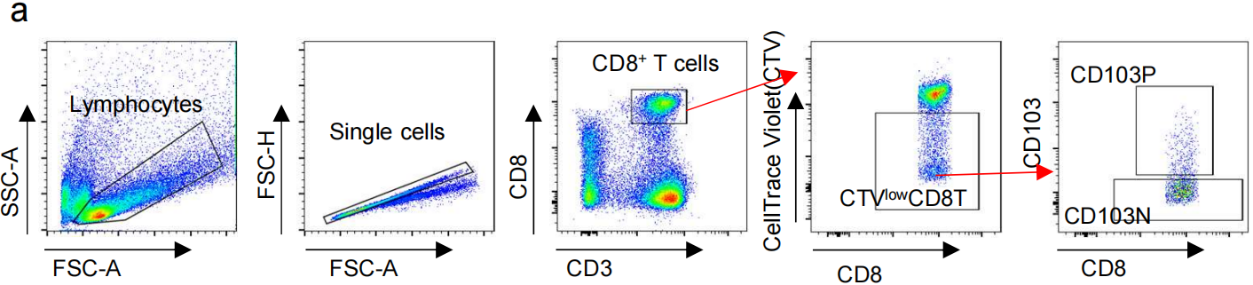
**

**Supplementary figure 2.** **CD8⁺ T cell proliferation in PBMCs upon stimulation with HCT116 cells.** (a) A representative flow cytometry plot illustrates the gating strategy used to identify CD103N and CD103P cells in proliferated CD8⁺ T cells with reduced CellTrace Violent levels (CTV^low^).
